# A 14-day ecological momentary assessment study on whether resilience and early family risk moderate daily stress and affect on cortisol diurnal slope

**DOI:** 10.1038/s41598-022-05277-w

**Published:** 2022-01-24

**Authors:** Natasha Yan Chi Tung, Yang Yap, Bei Bei, Linda J. Luecken, Joshua F. Wiley

**Affiliations:** 1 School of Psychological Sciences and Turner Institute for Brain and Mental Health, Monash University, 18 Innovation Walk, Melbourne, VIC 3800 Australia; 2 Department of Psychology, Arizona State University, Tempe, USA

**Keywords:** Psychology, Risk factors

## Abstract

This study examined whether resilience capacity moderates the association of daily perceived stress and affect with cortisol diurnal slope among relocated emerging adults. Relocated undergraduates (*N* = 98; aged 18–25 years) were recruited from three groups: Resilient, Vulnerable, and Control. The Resilient group required Risky Family Questionnaire (RFQ) scores ≥ 29 and Brief Resilience Scale (BRS) scores ≥ 3.6. The Vulnerable group required RFQ scores ≥ 29 and BRS scores ≤ 3. The comparison Control group required RFQ scores ≤ 21 and T-scores < 60 on PROMIS anxiety and depression symptoms. Mixed-effects models were used to test the unique associations of perceived stress, negative affect, and positive affect x group interactions (predictors) on diurnal cortisol slope (outcome) across 14 consecutive days. The Resilient group did not moderate the associations between daily stress or affect on cortisol diurnal slope. Instead, both the Resilient and Vulnerable groups with early family risk, showed a steeper diurnal slope unique to higher stress and a flatter slope unique to higher negative affect. Results suggest that riskier early family life was significantly associated with altered cortisol diurnal slope outcomes to stress (i.e., demand) and negative affect (i.e., distress). These associations were not attenuated by current resilience capacity.

## Introduction

Cortisol is a stress-sensitive steroid hormone regulated by the hypothalamic–pituitary–adrenal (HPA) axis that prepares the body to mobilize energy in managing threat (Ross et al., 2014). In humans, the cortisol diurnal rhythm is the daily circadian pattern of cortisol secretion peaking around 20–30 min after awakening followed by a decline throughout the day to its nadir 2 to 3 h after sleep onset^1^. Cortisol’s diurnal slope is derived from this decline from wake cortisol levels to pre-sleep cortisol levels, with a steady decline indicating healthy cortisol regulation^2^. Flattened cortisol diurnal slopes occur when there is lower wake cortisol levels or sustained elevation of cortisol levels at pre-sleep^3^. Flattened cortisol diurnal slopes are linked to poorer emotional and physical health such as cardiovascular diseases in a meta-analysis of 80 studies across all ages ranging from children to older adults^4^.

Chronic stress such as a risky early family background can influence the development and adult functioning of the HPA-axis^5^. Cortisol diurnal slope also responds to changes in individuals' experiences on a given day, notably in response to stress and affect^1^. Although daily stress and affect co-occur, they are conceptually distinct. Together, stress, negative affect (NA), and positive affect (PA) capture a range of daily experiences including situational demand (stress), negative experiences (NA), and positive experiences (PA)^6^. Stress and affect can have unique effects on cortisol, yet these are rarely separated in the literature^7,8^.

Individuals differ in their experience of and physiological response to situations depending on their psychological appraisal^9,10^. Individuals with high resilience capacity, defined as the trait of bouncing back from hardship^11^, evaluate challenges as manageable and respond better to stress. However, few studies have examined the role of resilience capacity, stress, and affect with cortisol diurnal slope on a daily basis, with most studies focussing on momentary cortisol levels or reactivity. To our knowledge, no studies have examined whether resilience capacity moderates the links of naturally occurring daily stress and affect with cortisol diurnal slope.

### Early family risk on cortisol diurnal slope

Research on the impact of childhood adversity as a type of chronic stress on cortisol diurnal slope has commonly found a flattened diurnal slope with elevated cortisol throughout the day among exposed children, attributed to a desensitization of the HPA-axis from over-engagement to recurring stressors^12^. These studies typically focussed on participants from backgrounds with highly adverse or abusive features, including children raised under institutional neglect^13^, with childhood trauma^14^, or with Child Protective Services involvement^15^. However, less is known about the impact of early family risk (defined as chaotic features occurring within general households) on cortisol diurnal slopes. For example, items measured in the Risky Family Questionnaire^16,17^ such as explosive arguments or neglectful parenting that increase risks for distress. Recent studies have addressed this gap by examining parenting features and found that poor parental monitoring^18^ and maternal neglect^19^ were also linked to flattened cortisol diurnal slope.

### Impact of daily stress and affect on cortisol diurnal slope

Daily stress refers to the daily demands of everyday living, including commuting, arguments, and work deadlines^20^. Daily stress and chronic stress impact cortisol diurnal slope differently but daily stress is rarely uniquely studied^21^. Yet, the daily variations in cortisol to daily changes are important to study as they exert downstream biological processes relevant to disease^1^. A naturalistic daily study showed cortisol diurnal slopes are steepened due to higher wake cortisol levels among older adults who reported higher frequency of stressors^20^. Overall, there is a lack of daily studies examining the relationship between daily stress and cortisol diurnal slope, with most examining *momentary cortisol level*s. These studies found momentary cortisol increases during the anticipation and experience of stressors^7,22^, and to higher perceived stress^23^. The association between higher momentary and wake cortisol levels with higher stress may be explained by the higher engagement of HPA-axis to meet the *demands* of the situation^24^. This adaptive response of diurnal cortisol activation if sustained can result in long-term physical and mental health issues^25^.

Beyond stress, NA and PA influence cortisol outcomes. Emotional responses to stressors, rather than chronic or daily stressors, predicted higher cortisol secretion *levels* in some^7,22,26^ but not all^20^ studies. One study found significant associations between NA and cortisol diurnal *slope* independent of stress^27^. However, affect may be influenced by antecedent stressors^20^. Hence, a separate assessment of affect and stress and simultaneous examination in one model is necessary to distinguish their unique influences on cortisol diurnal slope.

Higher NA levels are associated with flatter cortisol diurnal slope among adolescents^28,29^ and among adults^30^. The experience of sadness manifested in social withdrawal and behavioural inactivity may result in lower wake cortisol levels or the experience of anger and tension may result in higher pre-sleep cortisol levels. The few daily studies that examined PA and cortisol diurnal slope found steeper diurnal slopes with higher PA among midlife healthy adults^3^ and high school students^29^, but no impact of PA on wake cortisol levels. The steeper decline in cortisol slope in these studies not driven by a higher wake cortisol level was interpreted as indicative of healthy functioning.

In sum, current literature recommends a separate yet simultaneous examination of the unique effects of stress and affect on cortisol diurnal slope. While findings are limited, they indicate that higher stress, higher PA and lower NA are associated with a steepened cortisol diurnal slope.

### Resilience capacity on cortisol diurnal slope

To our knowledge, no studies have investigated if psychological resilience moderates daily stress/affect associations with cortisol diurnal slope. However, there are findings suggestive of the protective role of resilience or resilience-related constructs on cortisol metrics. Adults with higher psychological resilience (measured by Brief Resilience Scale) had a weaker association between perceived stress and hair cortisol *levels* over 3 months compared to those with low resilience^31^. High-risk individuals with high emotional regulation show minimal dysregulation in average basal cortisol across time^32^. Among children of parents with HIV, resilience (measured by Connor-Davison Resilience Scale) was associated with steeper cortisol diurnal slopes via less experienced stigma^33^. Taken together, there is evidence for hypothesising that psychological resilience buffers the dysregulation of cortisol diurnal slope in response to stress or exposure to risk.

## Aims and hypotheses

The simultaneous examination of the relations between resilience capacity, momentary stress, and affect with daily diurnal cortisol slopes remains underexplored. Further, resilience capacity should be most helpful in the presence of risk, which often is not captured in resilience studies^34^. Using retrospective reports of family risk as an index of prior adversity, our study recruited participants from three groups: (1) Resilient, including individuals who originated from risky families and reported high current resilience capacity, (2) Vulnerable, including individuals from risky families but with low reported current resilience capacity, and (3) Control, including individuals with low early family risk and average current symptoms of anxiety and depression. Participants were repeatedly assessed in their daily stress, affect, and cortisol at specific timepoints using ecological momentary assessments (EMA). EMA entails repeated, intensive sampling of respondents' current experiences (can be both objective and self-report assessments) while they are engaging with their typical daily routines^35^. All participants were emerging adults who relocated (i.e., moved) for tertiary studies; therefore, their daily experiences represent a known stressful transition period^36^.

We hypothesized that (1a) across the sample, a steeper, negative cortisol diurnal slope would be associated with higher perceived stress, higher PA, and lower NA, (1b) on average, the Resilient group would demonstrate a similar diurnal cortisol slope as the low-risk Control group whereas the Vulnerable group would demonstrate a flatter diurnal slope than the Resilient or the Control groups. Our study further explored whether the relation between stress and affect on diurnal cortisol slope is moderated in the Resilient group compared to the similarly high-risk Vulnerable group. Specifically, compared to the Vulnerable group, the Resilient group individuals were expected to have a weaker association between (2a) stress and diurnal cortisol slope, (2b) NA and diurnal cortisol slope, and (2c) PA and diurnal cortisol slope.

## Method

### Transparency and openness

We report how we determined our sample size, all data exclusions, and all measures included. Analysis code [https://doi.org/10.26180/14703843] and research materials [https://doi.org/10.26180/14593986.v1, https://doi.org/10.26180/14594238.v2] are available. Data will be made available on reasonable request and are planned for future public sharing in redacted form. The study and analysis plan were not pre-registered.

### Participants

A total of 98 international or interstate students aged 18 to 25 years old (*M*_*age*_ = 20.54*, SD*_*age*_ = 1.64) who moved interstate or overseas to commence tertiary studies in Melbourne, Australia completed the study between March 2019–June 2020. Data from 95 participants with viable cortisol samples were used. Supplementary Fig. 2 shows the participant flow chart and eligibility. Reporting follows the Strengthening the Reporting of Observational Studies in Epidemiology (STROBE) and Checklist for Reporting EMA Studies (CREMAS) reporting guidelines^37,38^ (Supplementary Table 2 and 3). *A-priori* power analysis conducted through G*Power^39^ indicated that with α = 0.05, 10 total predictors, and testing two predictors at once (appropriate as we have three groups), 75 participants, assuming a 75% compliance rate of 2 daily cortisol assessments across 14 days and intraclass correlation coefficients (ICCs) of 0.20 or 0.40 for cortisol provides 175–315 effective independent observations which provides 80% power to detect a medium effect size (Cohen’s *f*^*2*^ = 0.15) or a small-to-medium effect size (Cohen’s *f*^*2*^ = 0.05, roughly equivalent to a Pearson’s *r* = 0.20). More participants were recruited to allow for attrition and other aims and outcomes from the broader study.

#### Grouping

Participants were grouped based on their responses to the Risky Family Questionnaire (RFQ)^16,17^, Brief Resilience Scale (BRS)^11^, and PROMIS Anxiety and Depression scores^40^ completed at baseline. To maximize individual variability in this study, only participants who scored within the top and bottom tertile of family risk were invited to this daily study. A tertile split has similarly been used in other resilience studies among students^41^. The Resilient group was defined as RFQ ≥ 29 and BRS ≥ 3.6, corresponding roughly to the top tertile in our baseline sample of 380 participants. The Vulnerable group was defined as RFQ ≥ 29 and BRS ≤ 3, corresponding to roughly the top tertile RFQ in our baseline sample and below mean BRS. Research has shown that below average resilience is associated with below average well-being^42^ so our resilience cut off was chosen as approximately the mean BRS from our baseline data. The comparison Control group was defined as RFQ ≤ 21, approximately the bottom tertile in our population, and T-scores < 60 on PROMIS anxiety and depression symptoms, at most mild symptoms^40^ (Supplementary Fig. 1 and Fig. 2). The comparison Control group was selected to be a reference group representing individuals with low early family risk and currently with no more than average/mild levels of distress. As noted in Supplementary Fig. 2, participants with recent major life stressors were excluded, so the Control group can be interpreted as representing diurnal cortisol slopes of people without early life or current major stress and without any significant current distress—a likely mentally healthy group.

### Measures for grouping

#### Resilience

Resilience was measured by the 6-item Brief Resilience Scale (BRS)^11^, which assesses individuals’ ability to bounce back from stressful situations. Items, rated from 1 to 5, are averaged yielding a total score from 1 to 5, with higher scores representing higher resilience (α = 0.82). Our sample scored between 1–5 for resilience. A methodological review of resilience measurement scales^43^ gave the BRS not only a high-quality rating but also noted it was the only resilience measure that assesses individual’s ability to bounce back from stressful situations rather than the availability of protective resources.

#### Family risk

The Risky Family Questionnaire (RFQ)^16,17^ measures individuals’ perceptions of their family life during childhood (before 18 years old) to assess the degree of risk for physical, mental, and emotional distress experienced using 13 items. Participants indicate from 1 to 5 (sum score range 13–65) the extent that they felt loved, were mistreated, lived in a household that was chaotic and so on. Sample items include “How often would you say there was quarrelling, arguing, or shouting between a parent and one of your siblings?”. Higher scores reflect riskier family environment (α = 0.85) and our sample scored between 13–57 on family risk.

#### Anxiety and depression symptoms

Anxiety (α = 0.94) and depression (α = 0.93) symptoms were measured on separate 8-item PROMIS short-form scales by participants indicating the frequency (ranging from 1 = never to 5 = always) of when they have felt emotions and physiological reactions related to anxiety (e.g., “I felt fearful”, “My worries overwhelmed me”) and depression (e.g., “I felt worthless”, “I felt like a failure”). All PROMIS measure scores were converted to a T-score metric with a general population mean and standard deviation of 50 and 10, respectively. Scores of 55–60 indicate mild symptoms, 60–70 moderate and > 70 severe^40^. Our sample scored between 37–83 for anxiety and 38–81 for depression.

### Design and procedure

**Figure 1:**
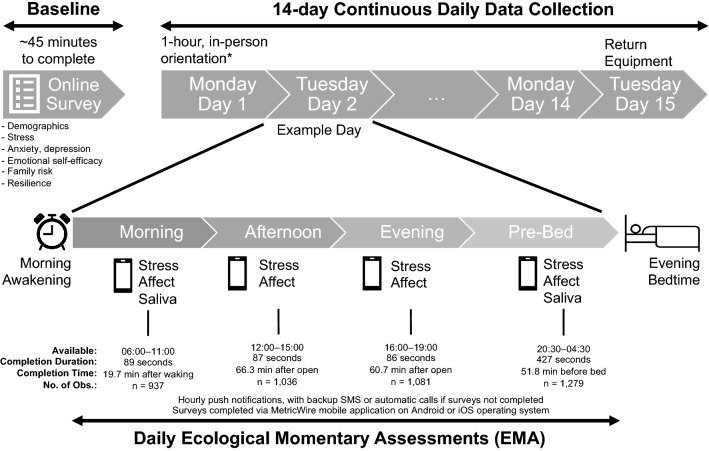
Study Procedure and Survey Completion. All participants started on Mondays and ended on Mondays, providing 10 weekdays and 4 weekend days. All surveys were closed outside their respective time windows to prevent retrospective reports. Stress and affect survey questions were identical over time to reduce participant burden, with some additional questions (not explored in this study) at the pre-sleep survey, resulting in longer completion time. Median completion time was preferred over mean time as it was possible that participants partially attempted surveys and completed them at a later time, leading to artificially longer completion duration. Participants received hourly push notifications (an average of 4 prompts), with backup SMS or automatic calls if surveys were not completed within time window. Surveys were completed via MetricWire mobile application on Android or iOS operating system. Participants attended a 1-h orientation session before starting the daily study, where they were trained in data collection protocol and provided with instruction manuals for completing surveys and saliva collection. *Participants who joined the study during COVID-19 restrictions were given the instructions through a recorded video and supported through teleconference platform Zoom. Subjects could reach a member of the research team by telephone if they had questions or problems during the sampling period. This period is expected to be a moderate stress period where university students are addressing daily hassles during an academic period of completing assessments or sitting for mid-semester examinations. All daily studies were timed to start during the academic semester and to avoid starting during holidays or break periods.

Monash University Human Research Ethics Committee (#17281) approved the study. Informed consent was obtained from all participants and the study was performed in accordance with relevant guidelines and regulations. Participants who completed the baseline questionnaire (~ 45 min) and met eligibility (per grouping cut-off scores as above) were invited to the daily study (Fig. 1; Supplementary Fig. 2). This daily study employed an intensive longitudinal observational design with daily repeated EMA across 14 days assessing participants’ stress and affect at 4 time points daily (Fig. 1) using a mobile application (MetricWire) on iOS or Android. Participants collected saliva samples using a synthetic cotton roll in Salivette tubes (SARSTEDT, Australia) immediately after waking and just before bedtime for 14-consecutive days. Participants recorded the date and time of saliva collection on the tube label, then attached a photo of the label to their morning and pre-sleep surveys. Recorded collection time was validated against the digital photo timestamp and self-reported wake/sleep times to monitor compliance. Participants were given a set of saliva collection compliance behaviour instructions (Supplementary section). They were encouraged to accurately report violations of these instructions, if any, in their pre-sleep surveys. Samples were removed if they did not meet collection criteria. Samples were stored in subjects’ home freezers until transport to the lab, where uncentrifuged samples were kept at either − 25 or − 80 °C freezers until analysis. The samples were then sent to the University of Dresden for assay analyses.

### Ethics approval

Monash University Human Research Ethics Committee (#17281) approved the study.

### Consent to participate and for publication

Flyer and explanatory statement including consent form https://doi.org/10.26180/14594001.

## Measures

### Salivary cortisol diurnal slope

Our study derived diurnal slopes from 2 cortisol samples on each of the 14 days, based on Segerstrom and colleagues’^2^ finding that diurnal slopes measured with this 2-sample approach (one at wake time and the other 9 pm) correlated 0.97 and 0.99 with diurnal slopes measured using 4 and 3 samples, and was better able to detect between- and within-person differences compared to more samples across fewer days. Raw cortisol values were natural log transformed to reduce skewness.

#### Stress

Perceived stress was measured using a single item “Since the previous survey, how stressful has your day been?” rated from 0 (Not at all stressful) to 10 (Very stressful).

#### Affect

Affect was measured using 14 items from the Positive and Negative Affect Schedule-Expanded version (PANAS-X) scale^44^. Participants were asked to rate how much they experience that affect since the previous survey or since wake for the first survey of the day based on “today”, on a 5-point Likert scale with 0 = “not at all” to 4 = “extremely”. Negative affect (7 items; e.g., “irritable”, “disgusted with self”, “sad”, “guilty”, “nervous”, “lonely”, “afraid”) had good between and adequate within reliability (ω_between_ = 0.89; ω_within_ = 0.62). Positive affect (7 items; e.g., “confident”, “relaxed”, “happy”, “enthusiastic”, “calm”, “cheerful”, “at ease”) had good between and within reliability (ω_between_ = 0.96; ω_within_ = 0.81).

#### Covariates

Covariates were selected a priori based on literature. Baseline covariates included age (years) and sex (male/female)^45^, subjective socioeconomic status^46^, race (coded as Asian/White/Others)^47^, nationality (international/interstate)^48^, English language acculturation (using the adapted Short Acculturation Scale for Hispanics to refer to participants’ native language instead of Spanish, with a score range of 1[low]–5[high]), time spent in Melbourne, COVID-19 period (pre [before Victoria lockdown 08/03/2020] vs during), body mass index (BMI; kg/m^2^ from self-reported height and weight) and alcohol consumption (coded abstainers/moderate/at-risk)^49^. Responses for sex and gender were equivalent in our data, hence combined into one variable. Daily covariates included number of daily stressors using an adapted self-report version of Daily Inventory of Stressful Events to which participants indicate yes/no to eight different types of stressors, for example an argument^50^. Other daily covariates included study day, month to account for seasonal variations in weather and progression in University semester, day of the week to account for differences in stress and affect during weekends vs weekdays^51^, compliance behavior violations (coded yes/no), and medication (coded taking vs not taking) during study period. There are no differences in results with including or excluding trait anxiety and depression^52^ as covariates.

### Analysis

Data were analyzed using R version 3.6.3^53^. Mixed models were estimated using lme4 v1.1–23 with restricted maximum likelihood. lmerTest v3.1–2 was used for degree of freedom and significance testing. All mixed models included a random intercept by participant to address non-independence. All covariates were included in all models. An identity link function was used with the outcome being natural log transformed salivary cortisol. Significance was set at α = 0.05, two-tailed. Visual model diagnostics were checked to evaluate assumptions. All assumptions were met. An example model equation is shown in the following. The outcome is natural logarithm transformed salivary cortisol for the *j*th participant at the *i*th assessment. The random intercept by participant is indicated by ${B}_{0j}$. Variables that are measured repeatedly are indicated by an *ij* subscript. Variables that are measured only once are indicated by only a *j* subscript. Affect and stress were explicitly decomposed into a within person (individual mean centered) and between person (individual mean) variables. Categorical variables were dummy coded prior to inclusion, indicated in parentheses.

$${ln\left(Cortisol\right)}_{ij}={B}_{0j}+{B}_{1-6}{DayofWeek}_{ij}\left(dummy coded\right)+ {B}_{7}{StudyDay}_{ij}+ {B}_{8-13}S{urveyMonth}_{ij}+ {B}_{14}N{egative Affect}_{ij} \left(within\right)+ {B}_{15}P{ositive Affect}_{ij} \left(within\right)+ {B}_{16}{Stress}_{ij} \left(within\right)+ {B}_{17}C{ortisolTime}_{ij}+ {B}_{18}{Age}_{j}+{B}_{19}{Born in Australia}_{j}+{B}_{20-22}{BMI Category}_{j}\left(dummy coded\right)+{B}_{23}{Sex}_{j}+{B}_{24}{Subject SES}_{j}+{B}_{25-26}{Race}_{j}\left(dummy coded\right)+{B}_{27}{Language Acculturation}_{j}+{B}_{28-29}{AUDIT Category}_{j}\left(dummy coded\right)+{B}_{30}{Anxiety Symptoms}_{j}+{B}_{31}{Depression Symptoms}_{j}+{B}_{32}{Average Number of Stressors}_{j}+{B}_{33}{COVID Period}_{j}+{B}_{34}{Negative Affect}_{j}\left(between\right)+{B}_{35}{Positive Affect}_{j}\left(between\right)+{B}_{36}{Stress}_{j}\left(between\right)+ {B}_{37-38}{Group}_{j}+{\varepsilon }_{ij}$$

Simple slopes were calculated for any significant interactions using high and low levels of perceived stress (4.24, 0.45), NA (2.06, 1.04) and PA (1.57, 3.64), determined based on the 90% and 10% percentiles in our sample. All analysis code and output is available at: https://doi.org/10.26180/14703843.

### Hypothesis 1: main effects of perceived stress, NA, PA, and main effects of perceived stress, NA, PA, and group on cortisol diurnal slope (two-way interactions)

This model tested whether between-person differences in daily perceived stress, NA, PA, and group were associated with cortisol diurnal slope via two-way interactions between daily stress/affect variables and time [wake or pre-sleep]. This model used the model equation shown previously, but also included four two-way interactions of timing of cortisol assessment x between person affect, stress, and group.

### Hypothesis 2: moderation of group on daily stress/affect-cortisol diurnal slope associations (three-way interactions)

This model tested the between-group differences in interactions with daily stress, NA and PA on cortisol diurnal slope (i.e., three-way interactions between groups [Resilient; Vulnerable; Control], daily stress/affect, and time [wake or pre-sleep] on cortisol levels). This model used the model equation shown previously, but also included three, three-way interactions: timing of cortisol assessment x between person affect/stress x group.

## Results

### Compliance

The 95 participants provided 4,333 surveys (74% completion rate) and 2,345 viable saliva samples (85% compliance rate) across 14 days, with omnibus test showing no significant differences across all groups (*p* = 0.92 for surveys and *p* = 0.39 for saliva samples respectively). In total, there were 71 (5.5%) saliva samples with reported violations of compliance behaviour, controlled for in analyses.

### Description of the sample

Table 1 shows the descriptive statistics and Supplemental Table 1 shows bivariate correlations. Most participants were female, of Asian descent, and were international students who spent less than a year in Melbourne. Participants exhibited the expected diurnal cortisol profile with average wake and pre-sleep cortisol of 11.32 and 1.29 nmol/l, which are comparable to healthy subjects in population-based studies^54^. None of the demographic variables or covariates were significantly associated with average cortisol levels except for age (*r* = − 0.28, *p* = 0.005) and time spent in Melbourne (*r* = − 0.22, *p* = 0.029). Participants reported average levels of daily stress, NA, and PA that were comparable to other young adult samples^55,56^. The Resilient and Control groups did not meaningfully differ^57^ on anxiety and depression symptoms, both showing lower symptoms than the Vulnerable group (Table 1).

**Table 1:** Descriptive statistics for demographic and daily variables (N = 95) by groups.

Variables	Overall (*N* = 98)	Control (*N* = 45)	Resilient (*N* = 19)	Vulnerable (*N* = 34)
**Demographic variables**				
Age (years)	20.48 (1.59)	20.39 (1.62)	20.24 (1.40)	20.73 (1.66)
Female (vs Male)	75 (78.9%)	33 (76.7%)	13 (72.2%)	29 (85.3%)
Single (vs In Relationship)	70 (73.7%)	32 (74.4%)	11 (61.1%)	27 (79.4%)
Subjective Socioeconomic Status (1–10)	5.56 (1.45)	5.72 (1.44)	5.72 (1.27)	5.26 (1.54)
**Race/ethnicity**^**+**^				
South Asian	13 (13.7%)	3 (7.0%)	4 (22.2%)	1 (2.9%)
Southeast Asian	44 (46.3%)	19 (44.2%)	10 (55.6%)	15 (44.1%)
East Asian	23 (24.2%)	14 (32.6%)	1 (5.6%)	1 (2.9%)
White/European/Anglo-Celtic	8 (8.4%)	4 (9.3%)	2 (11.1%)	8 (23.5%)
Others	7 (7.4%)	3 (7.0%)	1 (5.6%)	6 (17.6%)
**Nationality**				
International (vs Interstate)	88 (92.6%)	41 (95.3%)	15 (83.3%)	32 (94.1%)
Time Spent in Melbourne (years)	0.73 (0.95)	0.64 (0.85)	0.38 (0.52)^V^	1.02 (1.16)^R^
First-time leaving home (Yes or No)	68 (71.6%)	30 (69.8%)	11 (61.1%)	27 (79.4%)
English Language Acculturation (1–5)	3.82 (1.02)	3.35 (1.05)^RV^	4.66 (0.53)^CV^	3.97 (0.86)^CR^
Non-English Native Speaker (vs English Native Speaker)	65 (68.4%)	35 (81.4%)^R^	6 (33.3%)^CV^	24 (70.6%)^R^
Full-time student (vs Part-time)	93 (97.9%)	41 (95.3%)	18 (100.0%)	34 (100.0%)
Employed (vs Unemployed)	20 (21.0%)	8 (18.6%)	4 (22.3%)	8 (23.5%)
Co-parented upbringing (vs Single-parented upbringing)	88 (92.6%)	40 (93.0%)	16 (88.9%)	32 (94.1%)
Family Risk (13–65)	27.07 (8.66)	18.79 (1.73)^RV^	34.39 (5.16)^C^	33.68 (5.70)^C^
Resilience (1–5)	3.19 (0.76)	3.50 (0.55)^RV^	3.94 (0.23)^CV^	2.40 (0.43)^CR^
Anxiety symptoms (T-Score)	56.07 (9.09)	51.49 (4.29)^V^	52.28 (8.76)^V^	63.87 (8.63)^CR^
Depressive symptoms (T-Score)	52.75 (9.24)	49.04 (6.73)^V^	48.09 (5.09)^V^	59.92 (9.98)^CR^
Stress at Baseline (0–56)	25.45 (7.78)	21.51 (6.73)^V^	24.00 (4.39)^V^	31.21 (7.01)^CR^
Pre-COVID-19 period (vs during COVID-19 period)	69 (72.6%)	32 (74.4%)	13 (72.2%)	24 (70.6%)
**Daily Variables**		ICC		
Perceived Stress levels (*range* 0–10)				
Morning, *No. Obs.* = 937	1.39 (1.97)	.39 (61%)		
Pre-sleep, *No. Obs.* = 1279	2.41 (2.46)	.39 (61%)		
Negative Affect levels (*range* 1–5)				
Morning, *No. Obs.* = 936	1.36 (0.55)	.61 (39%)		
Pre-sleep, *No. Obs.* = 1275	1.49 (0.70)	.61 (39%)		
Positive Affect levels (*range* 1–5)				
Morning, *No. Obs.* = 936	2.65 (1.01)	.65 (35%)		
Pre-sleep, *No. Obs.* = 1279	2.63 (1.01)	.61 (39%)		
Cortisol levels (nmol/L)				
Morning, *No. Obs.* = 1142	11.32 (14.68)	.23 (77%)		
Pre-sleep, *No. Obs.* = 1203	1.29 (1.93)	.34 (66%)		

*ICC* Intraclass Correlations, *No. of Obs* Number of observations. Results are M (SD) for continuous variables and N (%) for categorical ones. Next to each variable, the scale (e.g., years) or possible range of a scale are shown in parentheses for continuous variables. For binary, categorical variables, the reference group is indicated in parentheses. Superscript V, R, and C represent significant differences with the marked group. ^+^ Chi-square test was used to examine significant differences in race by group and revealed no significant differences (*p* = .19).

### Association between cortisol diurnal slope with perceived stress, PA, and NA

There was a significant interaction effect for PA with cortisol diurnal slope (b [95% CI] = − 0.09 [− 0.17, − 0.00], *p* = 0.046) where higher levels of PA predicted a steeper, negative diurnal cortisol slope. No significant interaction was found for perceived stress (*p* = 0.23) or NA (*p* = 0.065) with cortisol diurnal slope (Table 2).

**Table 2:** 2-Way diurnal cortisol slope × stress/affect/group interactions and simple slopes.

	Stress	Negative affect	Positive affect	Group
**2-way Interaction: diurnal cortisol slope ×**				
Variable	− 0.05, *p* = .23 [− 0.12, 0.03]	0.20, *p* = .065 [− 0.003, 0.42]	− **0.09, *p* = .046** **[**− **0.17, **− **0.004]**	–
Vulnerable vs control	–	–	–	− 0.13, *p* = 0.14 [− 0.30, 0.04]
Vulnerable vs resilient	–	–	–	− 0.11, *p* = 0.27 [− 0.30, 0.08]
Resilient vs control	–	–	–	− 0.02, *p* = 0.80 [− 0.19, 0.15]
Simple Diurnal Slopes				
Low (10^th^ Percentile)	− **2.37, *p* < .001** **[**− **2.50, **− **2.23]**	− **2.51, *p* < .001** **[**− **2.62, **− **2.41]**	− **2.35, *p* < .001** **[**− **2.46, **− **2.24]**	–
High (90^th^ Percentile)	− **2.56, *p* < .001** **[**− **2.78, **− **2.35]**	− **2.29, *p* < .001** **[**− **2.46, **− **2.13]**	− **2.51, *p* < .001** **[**− **2.62, **− **2.41]**	–
Control	–	–	–	− **2.39, *p* < .001** **[**− **2.49, **− **2.28]**
Resilient	–	–	–	− **2.41, *p* < .001** **[**− **2.55, **− **2.27]**
Vulnerable	–	–	–	− **2.52, *p* < .001** **[**− **2.64, **− **2.39]**

Bold highlights *p* < .05. Estimates are unstandardized regression coefficients for interactions or simple slopes, followed by *p*-values, and 95% confidence intervals. All estimates are from linear mixed models with all covariates included.

### The differences in diurnal cortisol slope between groups

There were no significant differences in cortisol diurnal slope between all three groups (all *p* ≥ 0.14, Table 2).

### Differences in associations between stress and cortisol diurnal slope between groups

**Figure 2:**
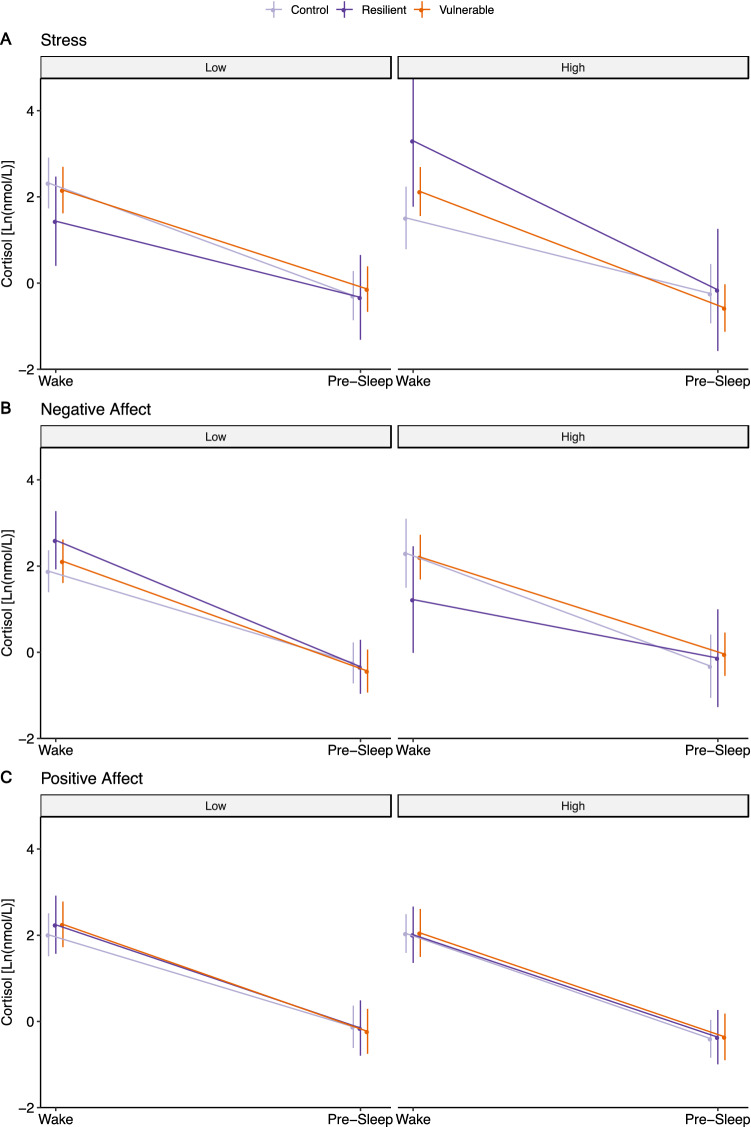
Three-way Interaction plot for the effects of Group and Perceived Stress/Affect predicting Cortisol Diurnal Slope. High and Low conditions of perceived stress (4.24, 0.45), negative (2.06, 1.04) and positive affect (1.57, 3.64) were determined based on the 90% and 10% percentile values of these variables within our sample.

Contrary to prediction, the Resilient and Vulnerable groups did not differ in their interactions with stress on cortisol diurnal slope, indicated by the absence of significant three-way interaction (*p* = 0.10) (Table 3). Compared to the Control group, both the Resilient (*p* < 0.001) and Vulnerable (*p* = 0.002) groups showed a stronger association between high stress and steeper diurnal slope (Table 3, Fig. 2A). Overall, groups significantly interacted with stress and cortisol diurnal slope at a small effect size *f*^2^ = 0.009, *p* < 0.001.

**Table 3:** 3-way diurnal cortisol slope × group × stress/affect interactions and simple slopes.

	Stress	Negative affect	Positive affect
**3-way interactions diurnal × group × variable**			
The vulnerable vs the control	− **0.29, *p* < .001** **[**− **0.47, **− **0.13]**	**0.73, *p* = *.*020** **[0.15, 1.36]**	0.20, *p* = .061 [− 0.01, 0.39]
The vulnerable vs the resilient	0.30, *p* = .10 [− 0.07, 0.65]	− **1.18, *p* = .029** **[**− **2.21, **− **0.10]**	0.03, *p* = *.*87 [− 0.32, 0.37]
The resilient vs the control	− **0.59, *p* = .002** **[**− **0.96, **− **0.21]**	**1.91, *p* = .002** **[0.74, 3.08]**	0.17, *p* = *.*30 [− 0.15, 0.48]
Simple 2-way interactions (diurnal × variable) by group			
The control	**0.20, *p* = .006** **[0.06, 0.34]**	− 0.45, *p* = .11 [− 1.02, 0.09]	− **0.16, *p* = .003** **[**− **0.26, **− **0.06]**
The resilient	− **0.39, *p* = *.*027** **[**− **0.74, **− **0.04]**	**1.46, *p* = *.*006** **[0.41, 2.47]**	0.009, *p* = .95 [− 0.29, 0.30]
The vulnerable	− **0.09, *p* = *.*043** **[**− **0.19, **− **0.01]**	**0.27, *p* = *.*032** **[0.04, 0.53]**	0.037, *p* = .68 [− 0.14, 0.21]
Simple diurnal slopes			
**Low variable levels**			
The control	− **2.61, *p* < .001** **[**− **2.83, **− **2.40]**	− **2.13, *p* < .001** **[**− **2.35, **− **1.91]**	− **2.14, *p* < .001** **[**− **2.31, **− **1.96]**
The resilient	− **1.77, *p* < .001** **[**− **2.35, **− **1.19]**	− **2.94, *p* < .001** **[**− **3.32, **− **2.56]**	− **2.40, *p* < .001** **[**− **2.75, **− **2.04]**
The vulnerable	− **2.30, *p* < .001** **[**− **2.52, **− **2.08]**	− **2.55,** ***p*** **< .001** **[**− **2.72, **− **2.38]**	− **2.48, *p* < .001** **[**− **2.67, **− **2.30]**
**High variable levels**			
The control	− **1.76, *p* < .001** **[**− **2.21, **− **1.30]**	− **2.62, *p* < .001** **[**− **3.07, **− **2.17]**	− **2.44, *p* < .001** **[**− **2.61, **− **2.28]**
The resilient	− **3.46, *p* < .001** **[**− **4.42, **− **2.50]**	− **1.36, *p* < .001** **[**− **2.15, **− **0.57]**	− **2.38, *p* < .001** **[**− **2.67, **− **2.08]**
The vulnerable	− **2.70, *p* < .001** **[**− **2.95, **− **2.45]**	− **2.25, *p* < .001** **[**− **2.47, **− **2.04]**	− **2.41, *p* < .001** **[**− **2.65, **− **2.17]**

Bold highlights *p* < .05. Variable in the table varies by column and is either stress, negative affect, or positive affect. Estimates are unstandardized regression coefficients for interactions or simple slopes, followed by *p*-values, and 95% confidence intervals. All estimates are from linear mixed models with all covariates included.

The steeper decline for the Resilient group at high stress was accompanied by a non-significant higher wake cortisol level at high stress compared to low stress (mean difference = 1.87, *p* = 0.12), which was also significantly higher than the Control group (*p* = 0.03). The steeper decline for the Vulnerable group was accompanied by a non-significant lower pre-sleep cortisol level at high stress compared to low stress (mean difference = − 0.44, *p* = 0.11). In contrast, the Control group showed a significant flatter diurnal slope with high stress (*p* = 0.006) accompanied by lower wake cortisol levels compared to low stress (mean difference = − 0.87, *p* = 0.09).

### Differences in associations between NA and cortisol diurnal slope between groups

Contrary to prediction, the Resilient group showed a significantly larger interaction effect with NA levels on cortisol diurnal slope compared to the Vulnerable (*p* = 0.029) and Control (*p* = 0.002) groups, indicated by significant three-way interactions (Table 3). Compared to both the Vulnerable and Control group, the Resilient group showed a significantly flatter diurnal slope at high NA (Table 3; Fig. 2B). The Vulnerable group also showed a significantly larger interaction effect (*p* = 0.020) and a flatter diurnal slope at high NA (*p* = 0.033) compared to the Control group. Groups significantly interacted with NA and cortisol diurnal slope at a small effect size (*f*^2^ = 0.006, *p* = 0.003).

The flatter slope of the Resilient group was accompanied by both non-significant lower wake cortisol (− 1.38, *p* = 0.09) and higher pre-sleep cortisol levels at high NA (0.20, *p* = 0.78) while the flattened slope of the Vulnerable group was accompanied by significant higher pre-sleep cortisol levels (0.39, *p* = 0.04). In contrast, the Control group showed no significant changes in cortisol diurnal slope by NA (*p* = 0.11).

### Differences in associations between PA and cortisol diurnal slope between groups

Contrary to prediction, the Resilient and Vulnerable groups did not differ in their interactions with PA levels on cortisol diurnal slope, shown by the absence of three-way interaction (*p* = 0.87) (Table 3). Both the Resilient and Vulnerable groups showed no significant changes in cortisol diurnal slope to PA (Table 3; Fig. 2C). The Control group showed a significant steeper negative diurnal slope at high PA (*p* = 0.003), although not significantly different than either the Vulnerable (*p* = 0.061) nor Resilient (*p* = 0.30) groups.

## Discussion

We found no overall changes in cortisol diurnal slope to higher stress and NA nor overall group differences in basal cortisol diurnal slope. When cortisol diurnal slope changes to stress and affect were examined by group, significant differences emerged. The Resilient group did not show the expected moderation effect on cortisol diurnal slope compared to their Vulnerable counterparts. Instead, the Resilient and Vulnerable groups both demonstrated a steeper diurnal cortisol slope with high stress compared to the Control group. Additionally, the Resilient group showed a flatter diurnal slope with high NA compared to the Control or Vulnerable groups.

Hypothesis 1 was partially supported. Higher PA was associated with a steeper, negative cortisol diurnal slope, consistent with previous research^3,29^. There were no significant associations between daily stress and NA with cortisol diurnal slope. The absence of a significant association for daily stress is consistent with similar null findings in other studies that controlled for affect as our study did^3,7,22^. Other studies also found null associations between NA with cortisol diurnal slopes or associations in the opposite direction to our hypothesis, requiring the consideration of arousal levels of NA or the interpretation of NA as harmful or not in measuring these associations^3,58^. Differences in our findings compared to existing literature also may be attributable to our collection of 2 saliva samples daily for 14 days, which better characterizes between-subject differences on cortisol slope^2^. Contrary to our prediction, the Vulnerable group did not exhibit an overall flatter cortisol diurnal slope than the Resilient or Control groups, perhaps because their early family risk was not at the severity or chronicity of abuse or maltreatment where flatter cortisol diurnal slopes previously have been shown.

Hypothesis 2 was not supported. The Resilient group did not show the hypothesized weakened interaction with stress/affect on cortisol diurnal slope. Instead, for high stress, both the Resilient and Vulnerable groups had steeper diurnal slopes compared to the Control group. The Resilient group’s steeper slope accompanied by higher wake cortisol levels may be explained by a greater engagement of the HPA-axis in approaching and managing demands^23^. The wake cortisol levels in the Vulnerable group did not change by stress, which may be explained by controlling NA (i.e., emotional distress) that often accompanies managing stress. Curiously, the Control group showed a flatter diurnal slope to high stress, contrary to existing literature^20^. The reason is unclear but our study examining the unique effects of stress, NA, and PA means perceived stress captures the experience of challenge/demand with neutral valence, perhaps explaining this distinct finding^59^.

At high NA levels, the Resilient group showed a significantly flatter cortisol diurnal slope compared to both the Vulnerable and Control groups. Our assumption was that the better psychological outcomes found among individuals with high resilience capacity compared to those with low resilience capacity^60^ would translate to healthier physiological outcomes in adverse situations. The flatter cortisol diurnal slope among the Resilient group compared to the similarly high-risk Vulnerable group may be interpreted as a heightened physiological sensitivity to emotional distress, reflecting a physiological cost for psychological adjustment^61^. These speculations need further exploration. Nevertheless, both the Resilient and Vulnerable groups showed flatter diurnal slope to high NA, which is indicative of the allostatic load of higher family risks resulting in heightened vigilance to threat (i.e., negative experiences) and therefore greater physiological reaction^62^ or greater susceptibility to the detrimental effects of emotional distress^63^. The non-significant association between NA and cortisol diurnal slope for the Control group may be explained by their perception of NA as non-threatening^58^.

The non-significant associations found for both the Vulnerable and the Resilient groups between PA and cortisol diurnal slope are not unique^7^. However, the steeper cortisol diurnal slope at high PA for the Control group suggests that the riskier family backgrounds of the Vulnerable and Resilient groups may predispose them to less physiological responsivity to PA. It is also possible that the different groups experienced different *arousal* levels of PA, which has been shown to affect cortisol diurnal slope. Specifically, high arousal PA has been linked to steeper cortisol slope^29^. However, our study did not differentiate between arousal levels. Nevertheless, these findings collectively suggest a unique pathway between resilience capacity and cortisol diurnal slope responses to negative vs positive emotions^64^. Further, there may be a difference in physiological sensitivity to affective valence between those from high and low risk families, such that the high risk Vulnerable and Resilient groups were more physiologically sensitive to demands and distress whereas the low risk Control group was more responsive to positive emotions.

Overall, the mostly similar cortisol diurnal patterns between the Vulnerable and the Resilient groups suggest that their common riskier early family backgrounds may be impacting on physiology more than current resilience capacity. This conjecture finds a basis in studies that proposed the programming of HPA-axis at sensitive early periods of development^65^. Additionally, the different cortisol diurnal slope changes to stress (i.e., demand) compared to NA (i.e., distress) support the need to look at these variables separately in future studies. Despite the groups not significantly differing in diurnal slope on average, meaningful differences emerged by stress and affect with the Control group showing opposite trends in cortisol diurnal slope responses when compared to their high risk Vulnerable and Resilient counterparts. These findings suggest that some of the conflicting results in cortisol research may be attributable to the lack of differentiation by resilience and family risk. Future cortisol studies may benefit from differentiating individuals by early risks and resilience capacity^66^.

## Limitation and strengths

This study had limitations. Menstrual cycle phase among females^45^ was not controlled. Despite carefully controlling for covariates that may impact stress and affect, we cannot rule out the influence of unmeasured aspects of the current psychosocial environment on cortisol outcomes such as quality of social contacts^67^. Moreover, cortisol levels only index part of physiological health^68^ and are not a pure estimate of HPA-axis functioning^69^. As with most daily studies, missing data was inevitable. Although the participants were grouped into high and low-risk families, the overall family risk is still low. Further, the sample size of each group varied, and family risk was measured retrospectively. Despite the shortcomings of retrospective self-report, existing findings suggest that the *appraisal* of early family life rather than objective occurrences impact outcomes more^70^. Our findings are not generalizable to different age group individuals nor those from abusive, high-risk families.

This study also had strengths. The compliance rate in our study was comparable to the 73–81% cohort compliance rate found in a meta-analysis of mobile EMA studies examining health-related and psychological constructs within a non-clinical adult population^71^. Also, we cross-checked reported saliva collection time against survey completion time and self-reported wake and sleep time, allowing higher confidence in our cortisol data accuracies^49^. Reviews on salivary cortisol collection have emphasized maximizing compliance while maintaining low participant burden as priorities^72^. We collected two saliva samples daily for 14 days, which should better characterize between-subject differences on cortisol slope^2^. Most prior studies collect more saliva samples across fewer days, some only on a single day^52^. The EMA design helped reduce retrospective recall bias. Given that the definition of resilience necessitates the presence of adversity, we measured the resilience of our participants against a context of early family life risk and current transitory stress by studying emerging adults at a developmental transition who moved at least interstate often internationally for tertiary studies. Most studies measure resilience based on self-report without the reporting of risks^34^. We also categorized participants from low-risk families into the Control group as demographically similar comparisons. However, our results could be even more confidently stated if we had an additional comparison group with low family risk and high emotional distress.

## Conclusions, future directions, and implications

Our daily study showed that early family life, even at the lower spectrum of risk, exerted significant differences in cortisol diurnal slope outcomes when experiencing high stress and distress that were not attenuated by current resilience capacity. These modest but daily physiological differences may accumulatively amount to substantial health impact such as poorer immunity and increased inflammation, as found in a meta-analysis^4^. Further, the examination of resilience, family risks, daily affect and stress and cortisol diurnal slope in a single paper is novel.

The larger flattening of cortisol diurnal slope among Resilient group to higher NA compared to Vulnerable and Control groups is novel. These results should be replicated in future daily studies with a focus on examining the contextual factors, such as whether the cortisol response is functionally adaptive based on the compatibility to the situational demand and coping behaviour^73^. Considering these contextual factors can better explain the differences in cortisol diurnal slopes by stress and affect between individuals of differing resilience and family risks. This increased understanding can inform the development of effective interventions for people with early family risk, regardless of their current psychological adjustment^74,75^.

## Supplementary Information

Supplementary Information.

## Data Availability

Data will be made available on reasonable request and is planned for public sharing as redacted dataset in the future.

## Code availability

Analysis code [https://doi.org/10.26180/14703843] and research materials are available at [https://doi.org/10.26180/14593986.v1training and manuals, https://doi.org/10.26180/14594238.v2 questionnaires].
