# Supplementary material for: A 14-day ecological momentary assessment study on whether resilience and early family risk moderate daily stress and affect on cortisol diurnal slope
Source: Sci Rep. 2022 Jan 24;12:1240. doi: 10.1038/s41598-022-05277-w (PMC8786880; doi:10.1038/s41598-022-05277-w)
Supplement: Supplementary file 1 — Supplementary Information. [file 41598_2022_5277_MOESM1_ESM.docx]

**Saliva Samples Exclusion Criteria**

Samples were removed if **1)** they were collected outside of ± 2hours or ± 4hours of their respective habitual wake or bedtime window period **2)** > 1-hour and > 4-hours discrepancy between the digital timestamp and written label for wake and sleep samples respectively and self-reported wake & sleep time **3)** > 30-mins after waking.

**Saliva Collection Compliance Behaviour instructions**

Participants were instructed not to 1) consume any major meals, alcohol, nicotine, caffeine, and/or medication 60-minutes before saliva collection, 2) brush teeth, chew gum, physically exert themselves, or drink water right before saliva collection.

# **Table 1**

*Correlations between Demographic Variables and Daily Variables*

| Variables | 1 | 2 | 3 | 4 | 5 | 6 | 7 | 8 | 9 | 10 | 11 | 12 | 13 |
| --- | --- | --- | --- | --- | --- | --- | --- | --- | --- | --- | --- | --- | --- |
| 1. Stress | - |  |  |  |  |  |  |  |  |  |  |  |  |
| 2. Positive Affect | -0.34*** | - |  |  |  |  |  |  |  |  |  |  |  |
| 3. Negative Affect | 0.76*** | -0.18 | - |  |  |  |  |  |  |  |  |  |  |
| 4. Cortisol | -0.09 | 0.05 | -0.02 | - |  |  |  |  |  |  |  |  |  |
| 5. Age | -0.05 | 0.06 | -0.09 | -0.28** | - |  |  |  |  |  |  |  |  |
| 6. Female | -0.07 | -0.09 | -0.07 | 0.14 | -0.14 | - |  |  |  |  |  |  |  |
| 7. Before COVID-19 | 0.08 | -0.01 | 0.09 | 0.03 | 0.05 | -0.20* | - |  |  |  |  |  |  |
| 8. Subjective Socioeconomic Status | -0.17 | 0.20* | -0.17 | 0.14 | -0.14 | 0.04 | 0.14 | - |  |  |  |  |  |
| 9. English Language Acculturation | 0.15 | -0.02 | 0.15 | -0.03 | 0.10 | -0.03 | 0.06 | 0.23* | - |  |  |  |  |
| 10. Time in Melbourne | 0.08 | -0.11 | 0.09 | -0.22* | 0.31** | 0.02 | - | 0.12 | -0.15 | - |  |  |  |
| 11. Nationality | -0.01 | 0.02 | 0.02 | 0.02 | -0.14 | 0.15 | -0.08 | 0.11 | 0.33*** | -0.12 | - |  |  |
| 12. Number of Daily Stressors | 0.60*** | -0.18 | 0.60*** | -0.05 | -0.03 | 0.07 | 0.06 | -0.10 | 0.14 | 0.26* | -0.02 | - |  |
| 13. Trait Anxiety | 0.62*** | -0.31** | 0.66*** | -0.04 | -0.07 | -0.06 | 0.11 | -0.27** | 0.18 | 0.05 | 0.02 | 0.42*** | - |
| 14. Trait Depression | 0.50*** | -0.27** | 0.59*** | -0.02 | 0.02 | 0.04 | 0.03 | -0.18 | 0.21* | 0.18 | 0.01 | 0.37*** | 0.80*** |

*Note.* * *p* < .05 ** *p* < .01 *** *p* < .001

#

**Table 2.**

*STROBE Statement—Checklist of Items that should be included in Reports of Observational Studies*

|  | Item No. | Recommendation | Page  No. | Relevant text from manuscript |
| --- | --- | --- | --- | --- |
| Title and abstract | 1 | (a) Indicate the study’s design with a commonly used term in the title or the abstract | Title page | A 14-Day Ecological Momentary Assessment Study |
|  |  | (b) Provide in the abstract an informative and balanced summary of what was done and what was found | 1 |  |
| Introduction | | | |  |
| Background/rationale | 2 | Explain the scientific background and rationale for the investigation being reported | 2-5 | The relation between resilience capacity, stress, and affect with daily diurnal cortisol slopes remains underexplored. |
| Objectives | 3 | State specific objectives, including any prespecified hypotheses | 5 |  |
| Methods | | | |  |
| Study design | 4 | Present key elements of study design early in the paper | 7 | This daily study employed an intensive longitudinal observational design with daily repeated EMA across 14 days assessing participants’ stress and affect at 4 time points daily using a mobile application (MetricWire) on iOS or Android. |
| Setting | 5 | Describe the setting, locations, and relevant dates, including periods of recruitment, exposure, follow-up, and data collection | 5-6 | completed the study between March 2019- June 2020. Data from 95 participants with usable cortisol samples were used. Supplementary Figure 1 shows the participant flow chart and eligibility. |
| Participants | 6 | (a) Cohort study—Give the eligibility criteria, and the sources and methods of selection of participants. Describe methods of follow-up  Case-control study—Give the eligibility criteria, and the sources and methods of case ascertainment and control selection. Give the rationale for the choice of cases and controls  **Cross-sectional study—Give the eligibility criteria, and the sources and methods of selection of participants** | 5, but mainly listed in Supplementary Figure 1. | Participants who completed the baseline questionnaire (~45 minutes) and met our selection criteria for daily study were invited to join (Figure 1; Supplementary Figure 2). |
|  |  | (b) Cohort study—For matched studies, give matching criteria and number of exposed and unexposed  Case-control study—For matched studies, give matching criteria and the number of controls per case |  |  |
| Variables | 7 | Clearly define all outcomes, exposures, predictors, potential confounders, and effect modifiers. Give diagnostic criteria, if applicable | 7-8 |  |
| Data sources/ measurement | 8* | For each variable of interest, give sources of data and details of methods of assessment (measurement). Describe comparability of assessment methods if there is more than one group | 7-8 |  |
| Bias | 9 | Describe any efforts to address potential sources of bias | 8 | Covariates were selected *a priori* based on literature. |
| Study size | 10 | Explain how the study size was arrived at | 6 | *A-priori* power analysis conducted through G* Power (Faul et al., 2007) ... |

| Quantitative variables | 11 | Explain how quantitative variables were handled in the analyses. If applicable, describe which groupings were chosen and why | 9 |  |
| --- | --- | --- | --- | --- |
| Statistical methods | 12 | (*a*) Describe all statistical methods, including those used to control for confounding | 9 |  |
|  |  | (*b*) Describe any methods used to examine subgroups and interactions | 9 |  |
|  |  | (*c*) Explain how missing data were addressed | 7 |  |
|  |  | (*d*) *Cohort study*—If applicable, explain how loss to follow-up was addressed  *Case-control study*—If applicable, explain how matching of cases and controls was addressed  ***Cross-sectional study*—If applicable, describe analytical methods taking account of sampling strategy** |  |  |
|  |  | (*e*) Describe any sensitivity analyses | n/a |  |
| Results | | | | |
| Participants | 13* | (a) Report numbers of individuals at each stage of study—eg numbers potentially eligible, examined for eligibility, confirmed eligible, included in the study, completing follow-up, and analysed | Supplementary Figure 2 |  |
|  |  | (b) Give reasons for non-participation at each stage | Supplementary Figure 2 |  |
|  |  | (c) Consider use of a flow diagram | Supplementary Figure 2 |  |
| Descriptive data | 14* | (a) Give characteristics of study participants (eg demographic, clinical, social) and information on exposures and potential confounders | 9 | (Under heading Description of the Sample) |
|  |  | (b) Indicate number of participants with missing data for each variable of interest | n/a, completion rate noted on p.10, attrition rate by group in flow chart Supplementary Figure 2 | The 95 participants provided 4,333 surveys (74% completion rate) and 2,345 usable saliva samples (85% compliance rate) across 14 days. |
|  |  | (c) *Cohort study*—Summarise follow-up time (eg, average and total amount) | n/a | n/a |
| Outcome data | 15* | *Cohort study*—Report numbers of outcome events or summary measures over time | n/a | n/a |
|  |  | *Case-control study—*Report numbers in each exposure category, or summary measures of exposure | n/a | n/a |
|  |  | ***Cross-sectional study—*Report numbers of outcome events or summary measures** | 9-10, 27 (Table 1) | n/a |
| Main results | 16 | (*a*) Give unadjusted estimates and, if applicable, confounder-adjusted estimates and their precision (eg, 95% confidence interval). Make clear which confounders were adjusted for and why they were included | 28-29  (Table 2 & 3) |  |
|  |  | (*b*) Report category boundaries when continuous variables were categorized | Supplementary |  |
|  |  | (*c*) If relevant, consider translating estimates of relative risk into absolute risk for a meaningful time period | n/a | n/a |

| Other analyses | 17 | Report other analyses done—eg analyses of subgroups and interactions, and sensitivity analyses | 10-12; 28-29 (Table 2 & 3) |  |
| --- | --- | --- | --- | --- |
| Discussion | | | | |
| Key results | 18 | Summarise key results with reference to study objectives | 12-15 |  |
| Limitations | 19 | Discuss limitations of the study, taking into account sources of potential bias or imprecision. Discuss both direction and magnitude of any potential bias | 14-15 |  |
| Interpretation | 20 | Give a cautious overall interpretation of results considering objectives, limitations, multiplicity of analyses, results from similar studies, and other relevant evidence | 12-16 |  |
| Generalisability | 21 | Discuss the generalisability (external validity) of the study results | 16 |  |
| Other information | |  | | |
| Funding | 22 | Give the source of funding and the role of the funders for the present study and, if applicable, for the original study on which the present article is based | Title Page | Wiley (1178487) and Bei (1140299) were supported by NHMRC fellowships. |

**Table 3.**

*An adapted STROBE Checklist for Reporting EMA Studies (CREMAS)*

|  |  | Item # | Checklist item | Page number reported | Relevant text from manuscript |
| --- | --- | --- | --- | --- | --- |
| Title | | | | |  |
|  |  | 1 | Include ecological momentary assessment in title and key words | Title Page | “A 14-Day Ecological Momentary Assessment Study” |
| Introduction | | | | |  |
|  | Rationale | 2 | Briefly introduce the concept of EMA and provide reasons for utilizing EMA for this study or topic of interests (eg, to examine time-varying predictors of unhealthy eating occasions in children’s daily lives) | 4-5 | The relation between resilience capacity, stress and affect with *daily* diurnal cortisol slopes remains underexplored (p.4)  Participants will be repeatedly assessed in their daily stress, affect and cortisol at specific timepoints using ecological momentary assessments (EMA) (p.5) |
| Methods | | | | |  |
|  | Training | 3 | Indicate if, and by what methods, training of participants for EMA protocol was used | 30 Figure1 | Participants attended a 1-hour orientation session before starting the daily study, where they were trained in data collection protocol and provided with instruction manuals for completing surveys and saliva collection. |
|  | Technology | 4 | Describe what technology, if any, was used. Include the following information: device (eg, mobile phone, portable computer), model (eg, Nexus 4, iPod), operating system (eg, Android, Windows), and EMA program name | 7 | ... using a mobile application (MetricWire) on iOS or Android. Participants collected saliva samples using a synthetic cotton roll in Salivette tubes (SARSTEDT, Australia)... |
|  | Wave duration | 5 | State the number of waves for the study (eg, 2 monitoring periods over the course of 1 year) | n/a | n/a |
|  | Monitoring period | 6 | State the number of days each wave of the study lasted, and how many weekdays versus weekend days | 30 Figure 1 | All participants started on Mondays and ended on Mondays, providing 10 weekdays and 4 weekend days. All surveys were closed outside their respective time windows to prevent retrospective reports. |
|  | Prompting design | 7 | Indicate the prompting strategy used for the study (eg, event-based, interval-based, or a combination of the two). If using interval-based strategy, indicate what type of schedule is used (eg, fixed, random, or hybrid interval) | 7; 30 Figure 1 | (Indicated in figure on p.30)  All surveys were closed outside their respective time windows to prevent retrospective reports. |
|  | Prompt frequency | 8 | Intended frequency of prompts per day. Break down by weekdays and weekend days if applicable | 30 Figure 1 | Participants received hourly push notifications (an average of 4 prompts), with backup SMS or automatic calls if surveys were not completed within time window. |
|  | Design features | 9 | Describe any design feature to address potential sources of bias (eg, reactivity) or participant burden (eg, EMA questions appearing in different orders) | 30 Figure 1 | Stress and affect survey questions were identical over time to reduce participant burden |
| Results | | | | |  |
|  | Attrition | 10 | Indicate participant attrition throughout the study; report attrition rates both by monitoring days and waves, if applicable | Supplementary Materials: Figure 2 |  |
|  | Prompt delivery | 11 | Report number of EMA prompts that were planned to be delivered. If possible, also report the number of EMA prompts that were actually received by participants and indicate reasons for why prompts were not sent out (eg, technical issues or participant noncompliance reason such as phone was powered off) | 30 Figure 1 | Participants received hourly push notifications (an average of 4 prompts), with backup SMS or automatic calls if surveys were not completed within time window. |
|  | Latency | 12 | Report the amount of time from prompt signal to answering of prompt | n/a | n/a |
|  | Compliance rate | 13 | Report total answered EMA prompts across all subjects and the average number of EMA prompts answered per person. Report compliance rate both by monitoring days and waves, if applicable. Indicate reasons for noncompliance, if known | 10 | The 95 participants provided 4,333 surveys (74% completion rate) and 2,345 usable saliva samples (85% compliance rate) across 14 days. In total, there were 71 (5.5%) saliva samples with reported violations of compliance behaviour, controlled for in analyses. |
|  | Missing data | 14 | Report whether EMA compliance is related to demographic or time-varying variables | 10 | ... with no significant differences across groups (*p* = .92 and *p* = .39 respectively). |
| Discussion | | | | |  |
|  | Limitations | 15 | Discuss limitations of the study, taking into account sources of potential bias when using EMA methods (eg, reactivity, use of technology) | 14-15 | As with most daily studies, missing data was inevitable. |
|  | Conclusions | 16 | Provide a general interpretation of results and discuss the benefits of using EMA (eg, improving understanding of daily behaviors) | 15-16 | The use of an EMA design helped reduce retrospective recall bias. |

**Figure 1**

*Grouping of participants*

*
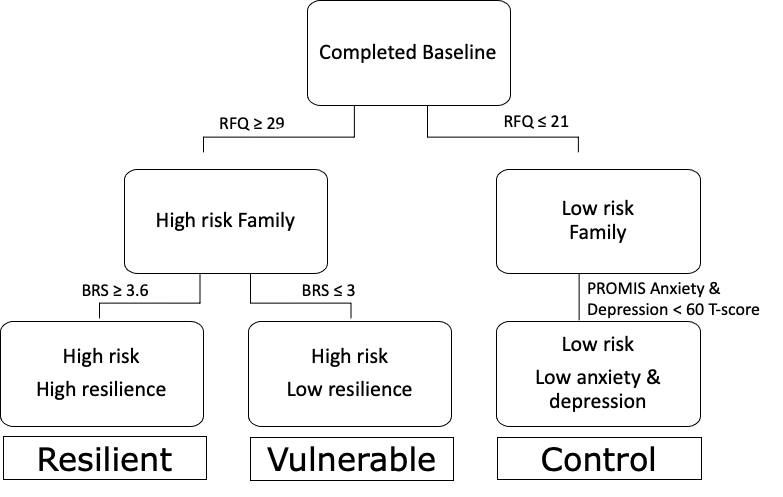
*

# **Figure 2**

*Participant Flow Chart*


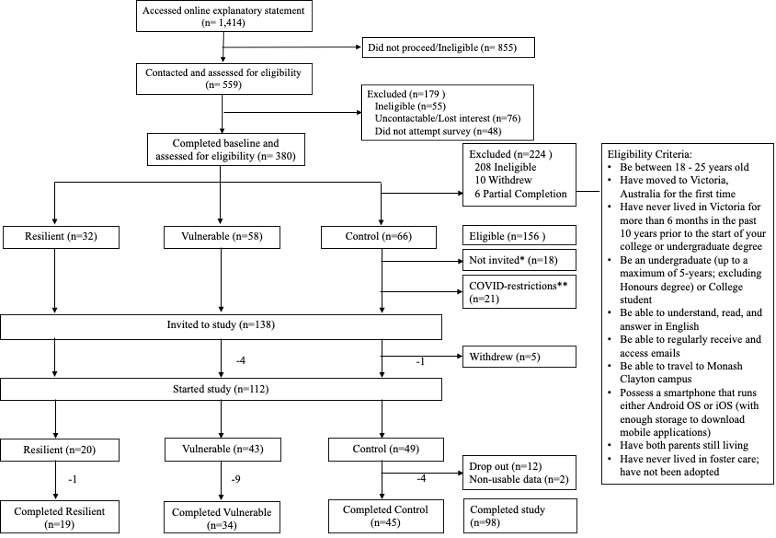


*Note*. *Not invited as daily study had discontinued due to COVID-19 restrictions ** Unable to travel to campus for daily study or participate due to COVID-19 interruptions to data collection. Participants first completed a baseline questionnaire assessing demographic information and other measures related to the overall study, with a subset meeting eligibility criteria and being invited for the Daily Phase used in the current study. Interested participants attended a 60-minute information session where they were trained in collecting their daily data and given written instructions.
